# Efficacy and Safety of Pioglitazone Monotherapy in Type 2 Diabetes Mellitus: A Systematic Review and Meta-Analysis of Randomised Controlled Trials

**DOI:** 10.1038/s41598-019-41854-2

**Published:** 2019-03-29

**Authors:** Fahmida Alam, Md. Asiful Islam, Mafauzy Mohamed, Imran Ahmad, Mohammad Amjad Kamal, Richard Donnelly, Iskandar Idris, Siew Hua Gan

**Affiliations:** 1 Human Genome Centre, School of Medical Sciences, Universiti Sains Malaysia, 16150 Kubang Kerian, Kelantan Malaysia; 2 Department of Haematology, School of Medical Sciences, Universiti Sains Malaysia, 16150 Kubang Kerian, Kelantan Malaysia; 3 Department of Medicine, School of Medical Sciences, Universiti Sains Malaysia, 16150 Kubang Kerian, Kelantan Malaysia; 4 Department of Family Medicine, School of Medical Sciences, Universiti Sains Malaysia, 16150 Kubang Kerian, Kelantan Malaysia; 5 King Fahd Medical Research Center, King Abdulaziz University, P. O. Box 80216, Jeddah 21589, Saudi Arabia; 6 Enzymoics, 7 Peterlee Place, Hebersham, NSW 2770 Australia; 7 Novel Global Community Educational Foundation, NSW, Australia; 8 Division of Medical Sciences & Graduate Entry Medicine, School of Medicine, University of Nottingham, Royal Derby Hospital Centre, Derby, UK; 9 School of Pharmacy, Monash University Malaysia, Jalan Lagoon Selatan, 47500 Bandar Sunway, Selangor Malaysia

## Abstract

Pioglitazone, the only thiazolidinedione drug in clinical practice is under scrutiny due to reported adverse effects, it’s unique insulin sensitising action provides rationale to remain as a therapeutic option for managing type 2 diabetes mellitus (T2DM). We conducted a systematic review and meta-analysis comparing pioglitazone monotherapy with monotherapies of other oral antidiabetic drugs for assessing its efficacy and safety in T2DM patients. Mean changes in glycated haemoglobin (HbA1c), and mean changes in fasting blood sugar (FBS) level, body weight (BW) and homeostasis model assessment-insulin resistance (HOMA-IR) were primary and secondary outcomes, respectively. Safety outcomes were changes in lipid parameters, blood pressure and incidences of adverse events. Metafor package of R software and RevMan software based on random-effects model were used for analyses. We included 16 randomised controlled trials. Pioglitazone monotherapy showed equivalent efficacy as comparators in reducing HbA1c by 0.05% (95% CI: −0.21 to 0.11) and greater efficacy in reducing FBS level by 0.24 mmol/l (95% CI: −0.48 to −0.01). Pioglitazone showed similar efficacy as comparators in reducing HOMA-IR (WMD: 0.05, 95% CI: −0.49 to 0.59) and increasing high-density lipoprotein level (WMD: 0.02 mmol/l, 95% CI: −0.06 to 0.10). Improved blood pressure (WMD: −1.05 mmHg, 95% CI: −4.29 to 2.19) and triglycerides level (WMD: −0.71 mmol/l, 95% CI: −1.70 to 0.28) were also observed with pioglitazone monotherapy. There was a significant association of pioglitazone with increased BW (WMD: 2.06 kg, 95% CI: 1.11 to 3.01) and risk of oedema (RR: 2.21, 95% CI: 1.48 to 3.31), though the risk of hypoglycaemia was absolutely lower (RR: 0.51, 95% CI: 0.33 to 0.80). Meta-analysis supported pioglitazone as an effective treatment option for T2DM patients to ameliorate hyperglycaemia, adverse lipid metabolism and blood pressure. Pioglitazone is suggested to prescribe following individual patient’s needs. It can be a choice of drug for insulin resistant T2DM patients having dyslipidaemia, hypertension or history of cardiovascular disease.

## Introduction

Type 2 diabetes mellitus (T2DM) is the most common chronic, metabolic disease whose prevalence is rapidly increasing worldwide. Insulin resistance (IR), the core metabolic defect contributes to the development of T2DM in approximately 92% of patients^1^. In IR condition, body cells mainly the peripheral adipose, muscle, and liver cells fail to respond properly to insulin signalling, resulting in decreased peripheral cells glucose uptake and increased hepatic glucose production^2^. Additionally, IR leads to impairment of insulin secretion by pancreatic β-cells. Hence, restoration of insulin sensitivity is the major treatment strategy for managing T2DM.

Thiazolidinediones (TZDs) are the only antidiabetic (AD) agents that function predominantly as insulin sensitisers in peripheral and hepatic tissues by binding to and activating nuclear peroxisome proliferator-activated receptor γ (PPARγ) expressed in those tissues. Among Food and Drug Administration (FDA) approved TZDs, troglitazone (Rezulin) was withdrawn from the market in 2000 due to severe hepatotoxicity whereas rosiglitazone (Avandia) has fallen out of favour owing to the controversy surrounding its cardiovascular (CV) safety^3^. Although FDA restricted the use of rosiglitazone in 2010, it later reversed the decision in 2013 after reanalysing the results of a multicentre randomised trial involving 4,447 T2DM patients where there was no reported increase in the incidence of myocardial infarction (MI) or CV death due to rosiglitazone^4^. However, restriction withdrawal on the rosiglitazone could not re-establish its previous reliability in clinical practice. Currently, pioglitazone (Actos) is the only available PPARγ agonist used for treating T2DM patients^5^.

Owing to IR, patients with T2DM are associated with a cluster of abnormalities such as dyslipidaemia, hypertension, increased expression of inflammatory mediators, decreased plasma adiponectin level, hypercoagulation and endothelial dysfunction. These abnormalities significantly increase the risk of developing atherosclerotic complications including stroke and MI, and has been associated with two to three-fold increase in CV mortality^6^. There are evidences where pioglitazone can modify these IR-mediated CV risk factors^7,8^, thereby exerting cardioprotective action^9^. In line with these observations, PPARγ are reported to reduce the plasma concentration of triglycerides (TGs) by increasing lipid accumulation in the adipose tissue. This effect decreases cardiac fatty acid uptake and oxidation, while increasing oxidative phosphorylation of glucose and lactate and therefore, provides CV safety by improving cardiac contractility^10,11^. In addition, compared with some AD agents, namely, sulfonylureas and insulin therapy, the use of pioglitazone either alone or in combination is associated with a lower risk of hypoglycaemia, a major risk factor for CV events^12^. Moreover, pioglitazone exert favourable effects in patients with nonalcoholic steatohepatitis^13^.

Despite these advantages, a host of adverse events, primarily body weight (BW) gain, peripheral oedema and congestive heart failure as well as controversy with the risk of bladder cancer has limit the use of pioglitazone in routine clinical practice. Thus, given its unique insulin sensitising effect, a risk-benefit analysis of pioglitazone treatment in patients with T2DM is crucial for determining its place in the current and future glucose-lowering treatment algorithm. This is particularly relevant given the current recommendation of individualisation of therapy in patients with T2DM, according to clinical and patient factors^14^. Therefore, we aimed to conduct a systematic review and meta-analysis comparing pioglitazone treatment as monotherapy with other AD monotherapies to confirm its efficacy and safety in T2DM patients.

## Methods

### Search strategy and selection criteria

This systematic review and meta-analysis was developed according to Preferred Reporting Items for Systematic Reviews and Meta-Analyses (PRISMA) Statement^15^ (Supplementary Table S1) with a predefined protocol registered under International Prospective Register of Systematic Reviews (PROSPERO) (identification number CRD42018088073). To identify relevant studies, seven electronic databases including Web of Science, Medline through PubMed, Embase, Scopus, Cochrane database (Cochrane Central Register of Controlled Trials), ClinicalTrials.gov and ScienceDirect were searched without restricting language and publication year up to May 30, 2018. During the electronic search, two themes of Medical Subject Headings (MeSH) terms (pioglitazone and diabetes) and related keywords were included, which were further combined with Boolean operators (‘AND’ and ‘OR’) using ‘Advanced’ and ‘Expert’ search options. The detailed search strategies for different databases are provided (Supplementary Text S1). Reviews, systematic reviews, meta-analysis, case reports, editorials, letters, erratum, comments, *in vivo* and *in vitro* studies were excluded. Duplicate records of electronic databases were removed by using the EndNote software (version X7.7). Two investigators (F.A. and M.A.I.) independently screened the titles and abstracts of the identified records. Full-text of relevant studies were retrieved to independently assess their compliance with the inclusion criteria. Any disagreements were resolved by consensus with a third investigator (I.I.). If full-text of relevant studies were unavailable online, either the corresponding authors or the first authors of those studies were contacted. Additionally, the reference lists of eligible publications were manually checked to find out the studies of interest.

Studies were regarded as eligible for inclusion if (1) they were randomised controlled trials (RCTs) conducted on T2DM subjects with no reporting of comorbid diseases or diabetes-associated complications, (2) they compared pioglitazone monotherapy with monotherapies of other FDA approved oral AD drugs, (3) the treatment duration was ≥12 weeks, (4) at least reported treatment effects on glycated haemoglobin (HbA1c) level, and (5) there was no prior history of T2DM patients treated with any AD drugs or discontinued monotherapy or combination therapy of AD drugs before starting the trials with or without washout period.

RCTs which compared pioglitazone monotherapy with injectable AD drugs or diet or exercise and also compared with AD drugs which have been discontinued (e.g. troglitazone) or debated (e.g. rosiglitazone) or under development (e.g. rivoglitazone) for T2DM treatment were excluded. RCTs where additional AD drugs were added as open-label rescue therapy in any treatment group during the trial period were excluded. In addition, duplicate publications representing subgroup analysis from the original publication as well as studies where overlapping of identical study subjects were observed with other included studies from similar research group were also excluded.

### Data extraction and quality assessment

Two investigators (F.A. and M.A.I.) independently extracted the data for all analyses. The following features were extracted from each study: first author and year (study ID), study location, trial type (multicentre or single centre), trial duration, sample size (males and females), comparator drugs name, HbA1c and fasting blood sugar (FBS) levels during enrolment, maintenance of lifestyle intervention during trials, number of patients (males and females) taking pioglitazone and comparator drugs along with their age, duration of diabetes and daily dose of drugs. Extracted information were then compared and any discrepancies such as unclear or missing data presentation were resolved by a consensus. If unresolved, requests for information were sent to corresponding or first authors of the respective studies for further clarifications.

To assess treatment efficacy, the mean change in HbA1c from baseline to study end was considered as the primary outcome. Depending on the reports of the selected studies, the mean changes in FBS, BW and homeostasis model assessment-insulin resistance (HOMA-IR) from baseline were considered as secondary outcomes. On the other hand, the change in blood pressure (BP) [systolic blood pressure (SBP) and diastolic blood pressure (DBP)], lipid parameters [low-density lipoprotein (LDL), high-density lipoprotein (HDL), total cholesterol (TC) and TGs] and records of adverse events were analyzed as safety outcomes. Predefined subgroup analyses were performed for the primary (changes in HbA1c) and secondary (changes in FBS) glycaemic outcomes based on specific comparator drugs, geographical location, trial duration, diabetes duration and pioglitazone dosage.

Heterogeneity of treatment effects between trials was assessed by using the χ² test (*p* < 0·10 indicates significant heterogeneity) and *I*²-statistic (degree of heterogeneity). The following thresholds of *I*^2^-statistic was considered to interpret heterogeneity: 0–25% (low heterogeneity), 26–75% (moderate heterogeneity) and 76–100% (substantial heterogeneity)^16^. If moderate or substantial heterogeneity was observed, we planned to perform sensitivity analyses for primary and secondary glycaemic outcomes to explore potential sources of heterogeneity excluding studies with overall high risk of bias (poor quality studies), open-label studies, multicentre studies and studies presented data in adjusted mean difference. We also planned to construct Galbraith plot if the source of heterogeneity remained unidentified after sensitivity analyses.

The methodological quality of each study was assessed by Cochrane Collaboration risk of bias tool^17^ based on the following criteria: random sequence generation, allocation concealment, blinding of participants and personnel, blinding of outcome assessment, incomplete outcome data and selective reporting. For each study, the overall risk of bias was regarded as high in the presence of high risk in any domain, low if all key domains (except random sequence generation and allocation concealment domain) were of low risk, and unclear in all other cases^18^. Publication bias was investigated via visual inspection of contour-enhanced funnel plot for primary and secondary glycaemic outcomes. Additionally, Begg’s and Egger’s tests were performed for quantitative publication bias analysis (*p* < 0.05 was considered significant)^19^. Results of publication bias were further validated by constructing trim and fill funnel plot.

For efficacy and safety measures, the mean differences in HbA1c, FPG, BW, HOMA-IR, SBP, DBP, LDL, HDL, TC and TG were calculated with standard deviations (SDs). They were assessed as continuous variables using weighted mean differences (WMD) and 95% confidence interval (CI). Study results which reported in standard error or 95% CI were converted into SDs. Safety measures reported as adverse events were assessed as dichotomous variables using risk ratio (RR) with 95% CI. Random-effects model was used for the meta-analyses. The results were considered statistically significant when *p* < 0.05. The forest plots were generated using the Review Manager (RevMan, version 5.3) software^20^. Illustration of Galbraith plot, funnel plots, and Begg’s and Egger’s tests were constructed in metafor package with R (version 3.4.1) and RStudio (version 1.0.153) software.

## Results

### Selection and inclusion of studies

**Figure 1:**
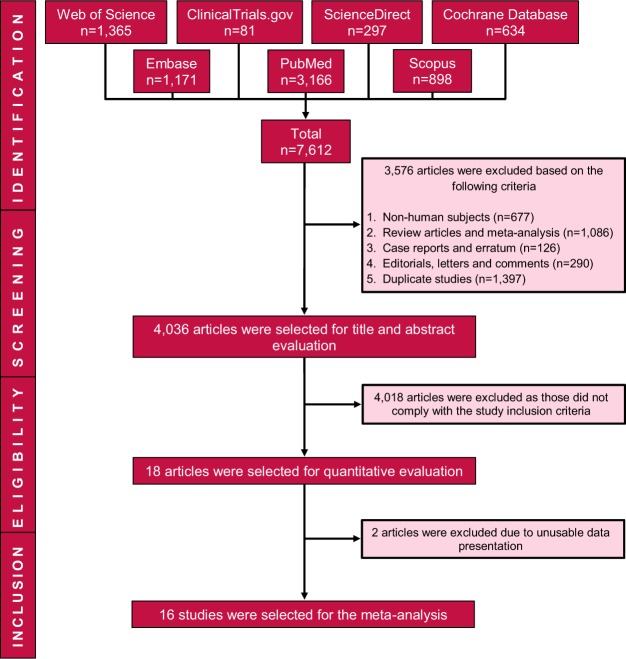
Flow chart of study selection process.

Based on the database search, a total of 7,612 articles were retrieved, from which 18 met the inclusion criteria^21–38^. Two studies were excluded as required data for meta-analyses couldn’t retrieved (Supplementary Text S2). The remaining 16 studies comprising 2,681 participants (1,503 males and 1,178 females, published between 2002 and 2017) were included in the meta-analyses. A flow chart of study selection process is illustrated in Fig. 1.

### Characteristics of included studies

Table 1 summarizes the major characteristics of the included studies. Most studies were conducted in Asia (six)^22–27^, followed by Europe (four)^21,28,29,31^, multinational (four)^32,33,35,36^ and North America (two)^30,34^. Only five studies were conducted for a minimum 12 weeks’ duration^21,24,25,35,36^. Nine out of 16 studies were multicentre. Mean age of participants randomised to pioglitazone and comparator drugs ranged from 45.1 ± 8.5 to 64.1 ± 8.5 years and 44.4 ± 10.6 to 65.1 ± 7.7 years, respectively. Five studies were conducted solely on naïve T2DM patients^21–24,27^, while in other studies, the mean diabetes duration of participants ranged from 2.2 ± 3.3 to 6.5 ± 4.7 years (pioglitazone) and 1.9 ± 3.1 to 6.4 ± 3.8 years (comparators). Pioglitazone was compared with metformin in six studies^21–24,26,27^, followed by sulfonylureas (SUs) in five studies^25,27,28,31,34^, dipeptidyl peptidase (DPP)-4 inhibitors in four studies^32,33,35,36^, α-glucosidase inhibitor (acarbose) in one study^29^ and meglitinide (repaglinide) in one study^30^. Among all studies, only one study compared pioglitazone to both metformin and a SU drug^27^.

**Table 1:** Major characteristics of the included studies.

No	Study ID	Study location, type	Study duration	Sample size (male/female)	Sample size (male/female)		Name of CPRs	Duration of diabetes (mean ± SD)		Age in years (mean ± SD)		Daily dose (mg/day)		HbA1c/FBS value during enrolment	Maintenance of lifestyle intervention during trials (YES/NO/NR)
					PIO	CPRs		PIO	CPRs	PIO	CPRs	PIO	CPRs		
1	Mori 2017	Japan, MC	3 m	58 (24/34)	29 (13/16)	29 (11/18)	Metformin	NR	NR	64.1 ± 8.5	65.1 ± 7.7	30 for male and 15 for female	750	NR/NR	NR
2	Esteghamati 2015a*	Iran, SC	12 w	88 (39/49)	46 (23/23)	42 (16/26)	Metformin	NDD	NDD	49.5 ± 2.0	49.4 ± 2.1	30	1000	NR/NR	NR
	Esteghamati 2015b*									53.5 ± 1.6	49.0 ± 1.7				
3	Esteghamati 2014a**	Iran, SC	3 m	81 (47/34)	42 (14/28)	39 (20/19)	Metformin	NDD	NDD	51.3 ± 7.9	50.0 ± 9.1	30	1000	≥6.5%/7.0 mmol/L	NR
4	Esteghamati 2014b**	Iran, SC	3 m	82 (36/46)	42 (19/23)	40 (17/23)	Metformin	NDD	NDD	51.8 ± 1.3	49.4 ± 1.3	30	1000	NR/NR	NR
5	Alba 2013	Mixed nations, MC	12 w	106 (51/55)	54 (23/31)	52 (28/24)	Sitagliptin	2.4 ± 1.4 y	2.4 ± 1.6 y	53.4 ± 7.8	54.6 ± 7.6	30	100	≥7–≤10% (drug naïve or ≥6.5–≤9% on treatment/≥7.2–\≤14.4 mmol/L	NR
6	Pérez-Monteverde 2011	Mixed nations, MC	12 w	492 (300/192)	248 (148/100)	244 (152/92)	Sitagliptin	3.5 ± 3.7 y	2.9 ± 2.8 y	51.7 ± 10.1	50.5 ± 10.9	15–30	100	≥7.5–≤12% />7.2–<17.8 mmol/L	NR
7	Hu 2010	China, SC	12 w	90 (45/45)	44 (21/23)	46 (24/22)	Glimepiride or gliclazide	6.5 ± 4.7 y	6.4 ± 3.8 y	52.6 ± 9.4	52.0 ± 9.1	15–45 (mean dose 25)	2–6 (mean dose 4) or 80–240 (mean dose 120)	≥7.0%/7.0–13.0 mmol/L	NR
8	Rosenstock 2010	Mixed nations, MC	26 w	327 (166/161)	163 (90/73)	164 (76/88)	Alogliptin	3.20 ± 3.74 y	3.23 ± 3.56 y	51.5 ± 10.7	52.6 ± 10.4	30	25	7.5–11%/NR	NR
9	Erdem 2008	Turkey, SC	12 w	44 (18/26)	21 (8/13)	23 (10/13)	Metformin	NDD	NDD	54.9 ± 7.8	55.1 ± 9.9	15–45	1000–2000	NR/NR	Yes
10	Cooper 2008	UK, SC	20 w	21 (16/5)	10 (8/2)	11 (8/3)	Gliben-clamide	~ 2.6 y	~ 2.4 y	56.0 ± 3.7	58.0 ± 2.6	45	5	<9%/NR	NR
11	Rosenstock 2007	Mixed locations, MC	24 w	315 (201/114)	161 (103/58)	154 (98/56)	Vildagliptin	2.2 ± 3.3 y	1.9 ± 3.1 y	52.4 ± 10.3	51.4 ± 10.8	30	100	7.5–11.0%/<15 mmol/L	NR
12	Perriello 2006	Italy, MC	12 m	283 (185/98)	146 (97/49)	137 (88/49)	Gliclazide	9.8 ± 5.4 y	8.5 ± 4.1 y	58.0 ± 8.0	59.0 ± 7.0	30–45 (mean dose 40)	80–320 (mean dose 84)	>7.5%/NR	NR
13	Rama-chandran 2004a***	India, SC	14 w	62 (46/16)	23 (17/6)	21 (15/6)	Metformin	NDD	NDD	45.1 ± 8.5	44.4 ± 10.6	15–30	250–850	8.5–11.0%/NR	Yes
	Rama-chandran 2004b***					18 (14/4)	Glimepiride		NDD		45.3 ± 10.3		1–2		
14	Tan 2004	Mexico, MC	52 w	244 (119/125)	121 (54/67)	123 (65/58)	Glimepiride	77.8 ± 79.2 m	81.2 ± 82.8 m	55.1 ± 8.0	55.7 ± 9.3	15–45 (mean dose 37)	2–8 (mean dose 6)	>7.5%–≤11% / NR	Yes
15	Jovanovic 2004	USA, MC	24 w	123 (67/56)	62 (31/31)	61 (36/25)	Repaglinide	6.1 ± 3.9 y	6.9 ± 6.0 y	56.2 ± 12.2	57.8 ± 13.1	30	10	>7.0%–<12%/NR	NR
16	Göke 2002	Germany, MC	26 w	265 (143/122)	129 (69/60)	136 (74/62)	Acarbose	57.0 ± 55.4 m	59.1 ± 50.3 m	58.9 ± 9.1	58.8 ± 9.1	45	300	7.5–11.5%/≥7.8 mmol/L	Yes

MS: Multicentre, SC: Single centre, NDD: newly diagnosed diabetes, NR: not reported, PIO: pioglitazone, CPRs: comparators, SD: standard deviation, m: months, w- weeks, y: years.

*Esteghamati 2015a and Esteghamati 2015b is same study, as results are divided into male and female patients, thus Esteghamati 2015a represents results of male patients and Esteghamati 2015b represents results of female patients.

**Esteghamati 2014a and Esteghamati 2014b are different studies but published in the same year.

***Ramachandran 2004a and Ramachandran 2004a is the same study, as PIO was compared with two different antidiabetic drugs, thus Ramachandran 2004a represents PIO vs metformin and Ramachandran 2004b represents PIO vs glimepiride.

### Risk of bias of the included studies

**Figure 2:**
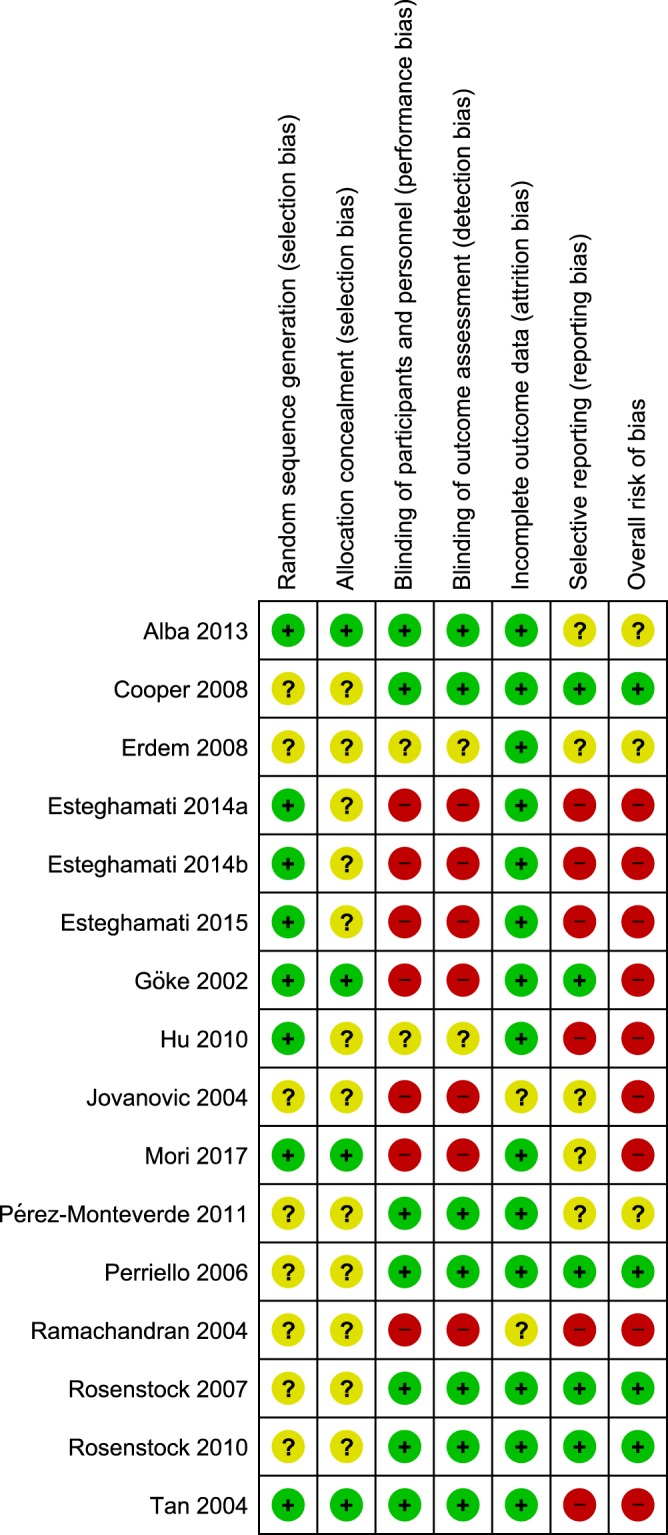
Risk of bias summary. Presentation of the risk of bias summary of the review author’s judgments about each risk of bias item for each included study. Studies in green or + are at low risk of bias.

Risk of bias assessment of the included studies were summarized in Fig. 2. Random sequence generation and allocation concealment were described adequately in eight^22–26,29,34,36^ and four^26,29,34,36^ of the 16 included studies, respectively. Seven studies were conducted as double-blinded^28,31–36^, 14^21–26,28,29,31–36^ studies reported the number of patients lost to follow-up while five^28,29,31–33^ studies mentioned the outcomes of interest in a pre-specified way. The overall risk of bias was low in four^28,31–33^, unclear in three^21,35,36^ and high in the remaining studies.

### Main results

#### Analysis of primary outcome

**Figure 3:**
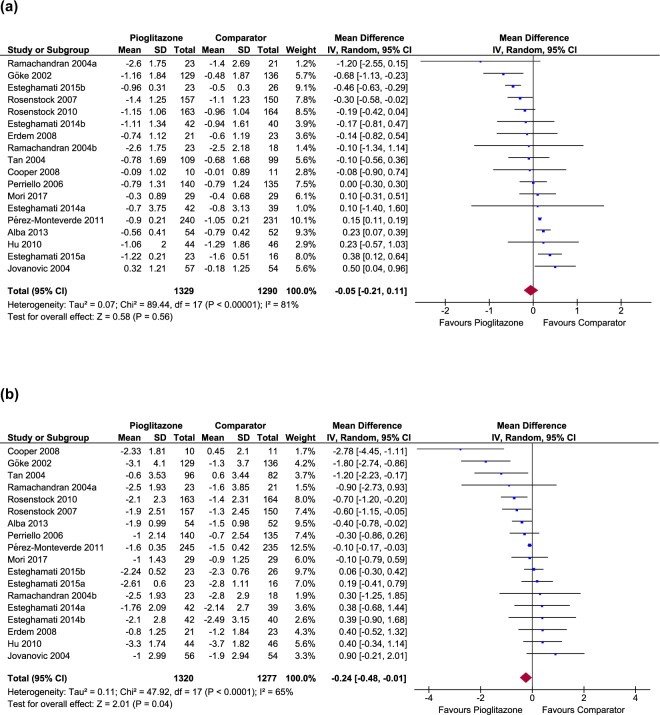
Forest plots showing effects of pioglitazone monotherapy versus comparator monotherapy on the primary (change in HbA1c) and secondary (change in FBS) glycaemic outcomes. Weighted mean difference in change from baseline in HbA1c (**a**) and FBS (**b**).

In primary outcome analysis of 16 studies, pioglitazone monotherapy reduced HbA1c by 0.05% with no significant difference (95% CI: −0.21 to 0.11, *p* = 0.56) and with substantial amount of heterogeneity (*I*^2^ = 81%, *p* < 0.00001) (Fig. 3).

#### Analyses of secondary outcomes

From secondary outcomes analyses, the effect of pioglitazone treatment was observed to be associated with decreased FBS level significantly by 0.24 mmol/l (95% CI: −0.48 to −0.01, *p* = 0.04) from the baseline with moderate heterogeneity (*I*^2^ = 65%, *p* < 0.0001) compared to the comparator AD drugs (Fig. 3), and associated with increased BW (WMD: 2.06, 95% CI: 1.11 to 3.01, *p* < 0.0001) without substantial heterogeneity (Supplementary Fig. S1). Both pioglitazone and comparator groups had similar effect on HOMA-IR (WMD: 0.05, 95% CI: −0.49 to 0.59, *p* = 0.86), associated with substantial heterogeneity (Supplementary Fig. S1).

#### Subgroup analyses

From subgroup analysis with specific comparator drugs, pioglitazone reduced HbA1c by 0.12% more than metformin (95% CI: −0.52 to 0.28, *p* = 0.57) and showed similar efficacy as SUs (WMD: −0.02, 95% CI: −0.24 to 0.21, *p* = 0.89) and DPP-4 inhibitors (WMD: 0.01, 95% CI: −0.19 to 0.20, *p* = 0.94). Though a significantly greater reduction by 0.68% in HbA1c was achieved with pioglitazone (WMD: −0.68, 95% CI: −1.13 to −0.23, *p* = 0.003) compared to acarbose, pioglitazone functioned less effectively than repaglinide (WMD: 0.50, 95% CI: 0.04 to 0.96, *p* = 0.03). In case of FBS reduction, pioglitazone was significantly more efficacious than DDP-4 inhibitors (WMD: −0.38, 95% CI: −0.70 to −0.06, *p* = 0.02) and acarbose (WMD: −1.80, 95% CI: −2.74 to −0.86, *p* = 0.0002), and also showed better efficacy than metformin and SUs, although the results were insignificant (Supplementary Fig. S2). Based on geographical location, HbA1c reduction by pioglitazone was more efficacious in European studies, followed by Asian and North American studies but pioglitazone was similarly efficacious as comparators in multinational studies, although the difference was not statistically significant in any of the groups. Based on the FBS analysis, efficacy of pioglitazone was statistically significant in decreasing FBS level in multinational studies (WMD: −0.38, 95% CI: −0.70 to −0.06, *p* = 0.02), followed by non-significant reduction in European, North American and Asian studies (Supplementary Fig. S3). Analysis of studies following trial duration revealed that patients receiving pioglitazone monotherapy had more HbA1c reduction than those receiving monotherapy of comparators when the treatment was prescribed for >12 weeks (WMD: −0.13, 95% CI: −0.31 to 0.05, *p* = 0.16). A similar scenario was observed with FBS where there was significant FBS reduction (WMD: −0.49, 95% CI: −0.92 to −0.05, *p* = 0.03) (Supplementary Fig. S4). In naïve T2DM patients, pioglitazone improved the glycaemic status by decreasing 0.16% of HbA1c level (95% CI: −0.61 to 0.28, *p* = 0.47) more than the comparators, whereas there was similar efficacy to comparators in patients with diabetes for long term (WMD: −0.02, 95% CI: −0.18 to 0.15, *p* = 0.85). In contrast, pioglitazone yielded significantly greater reduction of FBS (WMD: −0.49, 95% CI: −0.84 to −0.13, *p* = 0.007) in long term T2DM patients as compared to naïve patients (Supplementary Fig. S5). Comparison between pioglitazone dosage showed that 30 mg fixed-dose was similarly efficacious as the comparators (WMD: −0.01, 95% CI: −0.29 to 0.28, *p* = 0.96), whereas 45 mg fixed-dose showed a trend towards greater HbA1c reduction compared with other AD drugs (WMD: −0.48, 95% CI: −1.04 to 0.07, *p* = 0.09). Studies prescribing variable-dose of pioglitazone in an increasing level from low to moderate/high dose reported 0.14% less reduction in HbA1c than the comparators (95% CI: 0.11 to 0.18, *p* < 0.00001). In case of FBS reduction, the efficacy of pioglitazone 45 mg fixed-dose was significantly greater (WMD: −2.04, 95% CI: −2.86 to −1.21, *p* < 0.00001), followed by 30 mg fixed-dose and variable dose (15–45 mg) than comparators (Supplementary Fig. S6).

#### Sensitivity analyses

**Figure 4:**
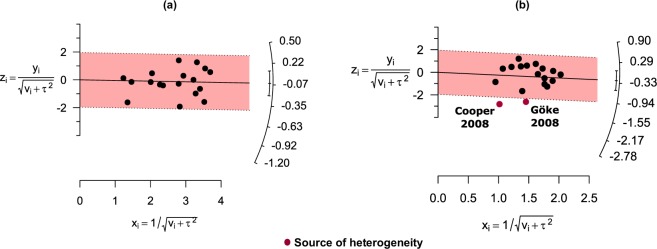
Galbraith plots illustrating the source of heterogeneity among included studies in HbA1c (**a**) and FBS (**b**) outcomes.

From sensitivity analyses, removal of multicentre (Supplementary Fig. S7), open-label (Supplementary Fig. S8), high risk of bias (Supplementary Fig. S9) and adjusted mean data presenting studies (Supplementary Fig. S10) from HbA1c and FBS main analyses failed to identify the responsible sources for substantial and moderate heterogeneity, respectively. Further investigation of HbA1c result with Galbraith plot was also unsuccessful whereas Galbraith plot construction following FBS findings identified two potential included studies^28,29^ as contributors for moderate heterogeneity (Fig. 4).

#### Publication bias assessment

**Figure 5:**
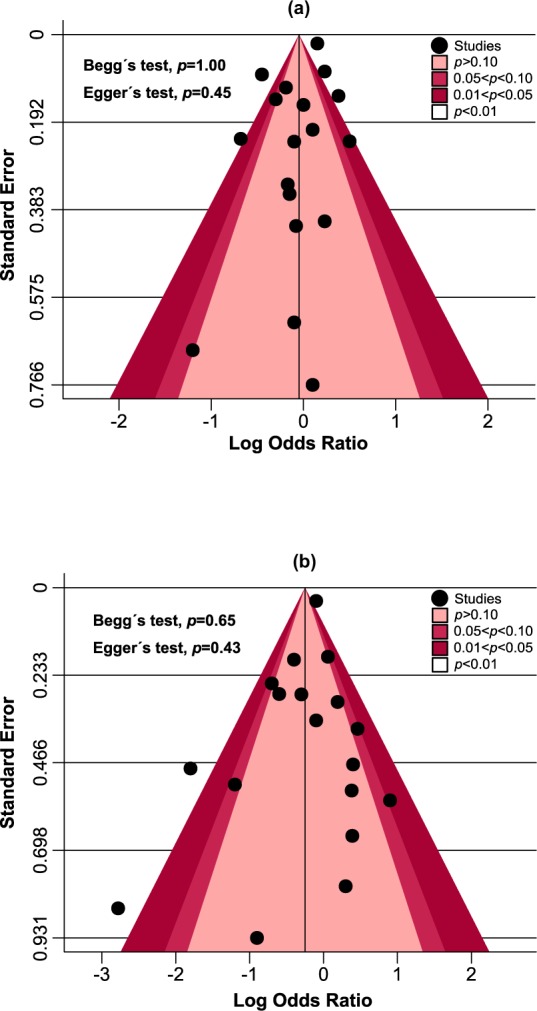
Contour-enhanced funnel plots of the included studies showing no evidence of publication bias in HbA1c (**a**) and FBS (**b**) outcomes.

**Figure 6:**
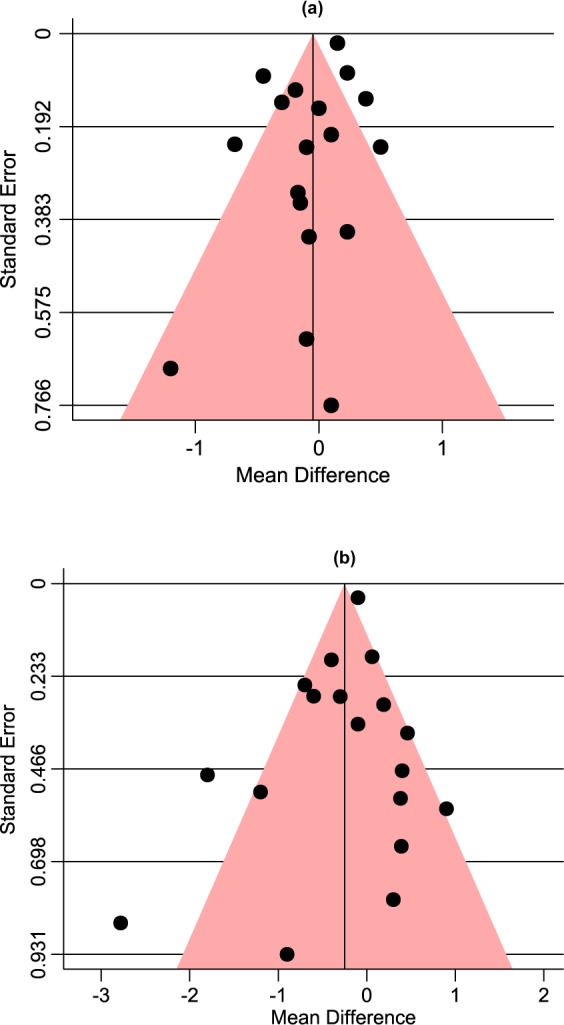
Trim and fill funnel plots showing absence of missing studies and verifying no evidence of publication bias in HbA1c (**a**) and FBS (**b**) outcomes.

Visual inspection of contour-enhanced funnel plots for HbA1c and FBS analyses showed no evidence of publication bias which were quantitatively validated by Begg’s (*p* = 1.00 and *p* = 0.65) and Egger’s tests (*p* = 0.45 and *p* = 0.43), respectively (Fig. 5). Further verification by trim and fill funnel plots showed no evidence of missing studies, thereby confirming the absence of publication bias (Fig. 6).

#### Analyses of safety outcomes

From safety outcomes analyses, improvements in BP was non-significantly greater (1.05 mmHg) in pioglitazone-treated patients than the comparators (95% CI: −4.29 to 2.19, *p* = 0.52). Among the lipid parameters, pioglitazone decreased TGs level by 0.71 mmol/l (95% CI: −1.70 to 0.28, *p* = 0.16) but couldn’t reduce the LDL and TC level as significantly as reduced by other comparative treatments. On the other hand, both groups exerted similar efficacy in increasing baseline HDL level without statistically significant difference (Supplementary Fig. S11). Among the reported adverse events, pioglitazone as initial monotherapy was not significantly associated with the risk of hypoglycaemia incidence (RR: 0.51, 95% CI: 0.33 to 0.80, *p* = 0.003), but significantly associated with risk of causing peripheral oedema in T2DM patients (RR: 2.21, 95% CI: 1.48 to 3.31, *p* = 0.0001) compared to the comparative monotherapy drugs. Besides, neither pioglitazone nor comparator drugs significantly contributed in developing upper respiratory tract infections (naspharingitis and sinusitis), nervous system disorders (headache, dizziness, syncope, cerebral ischaemia), musculoskeletal and connective tissue disorders (anthralgia, back pain, musculoskeletal pain), vascular disorders (arterial thrombosis and aortic stenosis), cardiovascular events, diarrhea, asthenia, abnormal liver functions, vomiting, nausea, colon cancer, breast cancer and non-cardiac chest pain (Supplementary Fig. S12). Interestingly, no incidence of bladder cancer was reported due to pioglitazone treatment.

## Discussion

Due to variations in mechanisms and sites of action, all existing AD drugs possess comparative differences in their glucose lowering efficacy. In our meta-analysis, pioglitazone monotherapy produced similar efficacy as other AD drugs in HbA1c reduction but greater efficacy with statistical significance in FBS reduction. Subgroup analysis with specific comparator drugs revealed pioglitazone to be a good choice of treatment in reducing HbA1c and FBS which was not inferior than metformin, SUs and DPP-4 inhibitors. This findings are supported by several previously published reports on patients with T2DM^39–44^. However, as only one study each comparing pioglitazone vs acarbose and pioglitazone vs repaglinide lead to opposite findings, it is difficult to judge which monotherapy was more efficacious. Subgroup analysis on geographical locations showed variable glycaemic response of T2DM patients with pioglitazone treatment, indicating variable drug response due to patients’ ethnic difference, age, sex, baseline weight and HbA1c^45^. Comparing studies in terms of duration of diabetes revealed a more pronounced efficacy of pioglitazone on FBS reduction in patients’ having long-term T2DM than in naïve T2DM patients’. It is plausible that prior AD treatment of a portion of patients before enrollment might have influenced the efficacy of pioglitazone. However, this scenario was not observed in the context of HbA1c reduction, therefore warranting further investigations. In subgroup analysis following trial duration, pioglitazone was efficacious in reducing HbA1c and FBS levels than comparators when the trials were conducted for >12 weeks duration suggesting a slower onset action of pioglitazone to give maximal effect. A few long-term (52-week) studies evaluating pioglitazone monotherapy also supported the sustained antihyperglycaemic effect of pioglitazone^34,44^. Subgroup analysis based on dosage revealed a higher efficacy of fixed-dose pioglitazone in improving glycaemic response than variable-dose which again suggests the gradual increase in therapeutic action by pioglitazone when given at a fixed-dose than in variable-dose.

In line with earlier findings in T2DM patients^39,46^, we also observed significant mean increase in patients’ BW (2.06 kg) following pioglitazone use. It is believed that increased BW with pioglitazone is due to fluid retention and fat accumulation in the body. Although previous studies reported BW gain in a higher rate, a few but contradictory findings exist where the use of pioglitazone without or with lower BW gain were also reported^5,41,47^. Nonetheless, BW gain is a major problem among pioglitazone users, which may limit its utility. However, numerous studies have shown the benefits of adjuvant strict dietary restriction with exercise intervention to attenuate pioglitazone-induced BW gain^48–50^.

The HOMA-IR method is widely used for assessing insulin resistance in clinical trials and epidemiological studies, improvement of which indicates enhanced insulin sensitivity. Since pioglitazone is an insulin sensitiser which improves insulin sensitivity by acting on peripheral and liver cells; it is anticipated that pioglitazone would improve HOMA-IR compared with comparator^37,51^. In this meta-analysis however, pioglitazone had similar efficacy as comparators on HOMA-IR. However, interestingly detailed analysis of individual studies revealed that studies (n = 3) conducted in Iran^22–24^ appear to favour the comparators rather than pioglitazone indicating the possible influence of genetic makeup in the pharmacodynamics of pioglitazone. Apart from the Iranian studies, other studies (n = 7) favoured pioglitazone with statistical significance (*p* = 0.05).

Consistent with previous observations, this meta-analysis confirmed the positive influence of pioglitazone on the lipid profile of T2DM patients with significant decrease in TGs and increase in HDL (although mean HDL increase was similar to comparators)^43,44,51–55^. Several studies have reported the effect of pioglitazone in increasing TC and LDL^43,44,54,55^, but pioglitazone appeared to be associated with TC and LDL reduction in this meta-analysis which was also supported by other evidences^51–53^. It is plausible that variations in treatment duration, pioglitazone dosage as well as patients’ compliance to pioglitazone are responsible for the contradictory findings of individual studies. Large number of clinical studies have reported pioglitazone as a good regulator of BP^53,55^. Our meta-analysis results also support the contribution of pioglitazone in reducing BP, particularly SBP, in patients with T2DM, although changes in BP from baseline were not significantly associated with pioglitazone use. Since hypertension is frequently diagnosed as a co-morbidity in patients with T2DM which could lead to long-term vascular and renal complications, BP-lowering efficacy of pioglitazone may be helpful in preventing the development of hypertension and its associated complications in T2DM patients.

Similar to prior studies^44,54^, the incidences of oedema were significantly higher with pioglitazone, while the incidences of hypoglycaemia were significantly lower than comparators. Although limited number of studies were included in these analyses, the presence of low heterogeneity indicates reliability of the results. The low hypoglycaemic risk associated with pioglitazone monotherapy was reported to be beneficial for T2DM patients with CV disease, especially in preventing mortality after severe hypoglycaemia^56^. Two meta-analyses investigated the association of pioglitazone with CV risk reported pioglitazone with no relevant effect on CV events among a diverse population of diabetes patients, significantly lower risk of death and reduced all-cause mortality, thereby further supporting our meta-analysis results^57,58^. Despite the favourable effect, pioglitazone treatment in patients with underlying heart disease may be harmful since pioglitazone-mediated peripheral oedema can progress into congestive heart failure. Apart from the aforementioned adverse events, analysis of other reported adverse events during pioglitazone treatment did not reach statistical significance due to insufficient included studies and therefore, these results were not able to evaluate the safety profile of pioglitazone. However, the incidence of bladder cancer during pioglitazone treatment was not reported in any of the included studies supporting the conclusion of a recent meta-analysis^59^ which suggested that other factors but not pioglitazone may contribute to the risk of bladder cancer.

Although a substantial and moderate amount of heterogeneity was noted in both HbA1c and FBS analyses respectively, sensitivity analyses could not explain the sources of heterogeneity based on the quantitative analysis (*I*^2^). Therefore, none of the factors analysed for sensitivity contributed to the heterogeneity. Even by using the Galbraith plot, responsible studies causing the substantial heterogeneity in HbA1c analysis were unidentified. However, the unidentified source of heterogeneity could potentially be attributed to variation among included trials regarding ethnicity of participants, dosing of comparators in the control group, types of diet and exercise maintained along with AD drugs, patients’ baseline characteristics such as baseline HbA1c and varying use of AD drugs before randomisation. We could not explore the influence of these factors through sensitivity analyses due to lack of relevant data. On the other hand, Galbraith plot revealed two contributing studies^28,29^ for the heterogeneity of FBS results. Since both studies utilised pioglitazone 45 mg fixed-dose and caused greater reduction of FBS from baseline than other studies, it is plausible that this factor is the source of heterogeneity.

This systematic review and meta-analysis has several notable strengths. First, to the best of our knowledge, it is the first systematic review and meta-analysis assessing the efficacy and safety of pioglitazone monotherapy against monotherapy of other AD drugs for treating patients with T2DM. Second, a comprehensive and robust literature search without year and language restriction were conducted across seven electronic databases following a standard methodology. Third, our inclusion criteria ensured that only FDA approved AD drugs currently in use in clinical practice were included for the analysis. We accepted only RCTs conducted for at least 12 weeks’ duration so that HbA1c test during the follow-up reflects the treatment efficacy on the average glucose levels of the previous three months. Fourth, no publication bias was observed from both visual (contour-enhanced funnel plot) and quantitative analyses (Begg’s and Egger’s tests). Additionally, the absence of missing studies from trim and fill funnel plot analysis further confirms that we were unlikely to have missed studies that could have altered the meta-analysis findings. Fifth, five different subgroup analyses have been conducted to find out the possible factors contributing to the efficacy of pioglitazone monotherapy on HbA1c reduction comparing to other drugs.

Despite these strengths, we do acknowledge certain limitations. First, the result of this meta-analysis is represented by a small sample size (n = 2,681) from only 16 RCTs. Second, we had to exclude two potential studies due to lack of data representation. We couldn’t retrieve the required information even after requesting the corresponding and first authors of those studies. Third, 9 out of 16 studies were judged as high methodological risk in overall bias assessment, which was contributed by RCTs with open-label study design and RCTs with reporting bias. Fourth, a substantial level of heterogeneity in HbA1c analysis was noted. We couldn’t identify the source of heterogeneity even after conducting different types of sensitivity analyses as well as constructing Galbraith plot. Fifth, none of the RCTs except one study^34^ were designed to evaluate the long-term efficacy and safety of pioglitazone monotherapy on T2DM patients. Also, a substantial number of studies did not report on adverse events possibly because they were not designed to evaluate such endpoint and not endowed with a sufficiently long study duration.

## Conclusions

Based on the findings of this meta-analysis, we concluded that pioglitazone monotherapy showed overall favourable risk-benefit balance. Specifically, pioglitazone is an effective treatment option in managing T2DM patients due to its potential of ameliorating hyperglycaemia, adverse lipid metabolism and BP. Improvement of these CV risk factors is crucial in terms of CV protection and stroke prevention in T2DM patients. Pioglitazone monotherapy can also be used as an alternative to metformin monotherapy if metformin cannot be tolerated or as a combination therapy if metformin alone fails to achieve target HbA1c level. Since hypoglycaemia is recognized as a potential cause of death^60^, particularly due to cerebral damage, the low hypoglycaemic risk of pioglitazone over other AD drugs will be advantageous in preventing mortality in T2DM patients. However, development of oedema and BW gain due to pioglitazone cannot be ignored. Hence, it is suggested to avoid pioglitazone treatment in patients with previously diagnosed heart failure. Since approximately >90% of T2DM patients are obese, pioglitazone-mediated BW gain can be ameliorated by lifestyle intervention including nutrition therapy and regular physical activity. Patients should be followed-up to monitor BW gain, since sudden, uncontrolled BW gain may be an indication of new onset heart failure. As oedema and weight gain is dose-dependent, low-dose of pioglitazone which proved as similarly efficacious as standard-dose in improving glucose and lipid metabolism^61^, could possibly be used as an alternative treatment. Additionally, combination of pioglitazone with FDA approved anti-obesity drug can be explored on T2DM obese patients. Whether pioglitazone increases the risk of bladder cancer in T2DM patients remains unclear, but no signal for this adverse event was observed in the meta-analysis. Since pioglitazone is the only insulin sensitiser among existing AD drugs and is the only TZD currently in use, we believe that the evidence from this meta-analysis support the ongoing role of pioglitazone in managing patients with T2DM. Furthermore, RCTs with pioglitazone monotherapy are suggested for weighing its long-term benefits and risks in T2DM patients.

## Supplementary information

Supplementary information
