# Supplementary material for: Efficacy and Safety of Pioglitazone Monotherapy in Type 2 Diabetes Mellitus: A Systematic Review and Meta-Analysis of Randomised Controlled Trials
Source: Sci Rep. 2019 Mar 29;9:5389. doi: 10.1038/s41598-019-41854-2 (PMC6441028; doi:10.1038/s41598-019-41854-2)
Supplement: Supplementary file 1 — Supplementary information [file 41598_2019_41854_MOESM1_ESM.docx]

**Safety and Efficacy of Pioglitazone Monotherapy in Type 2 Diabetes Mellitus: A Systematic Review and Meta-Analysis of Randomised Controlled Trials**

Fahmida Alam^1,^ *, Md. Asiful Islam^2^, Mafauzy Mohamed^3^, Imran Ahmad^4^, Mohammad Amjad Kamal^5, 6, 7^, Richard Donnelly^8^, Iskandar Idris^8^, Siew Hua Gan^9,^ *

^1^Human Genome Centre, School of Medical Sciences, Universiti Sains Malaysia, 16150 Kubang Kerian, Kelantan, Malaysia

^2^Department of Haematology, School of Medical Sciences, Universiti Sains Malaysia, 16150 Kubang Kerian, Kelantan, Malaysia

^3^Department of Medicine, School of Medical Sciences, Universiti Sains Malaysia, 16150 Kubang Kerian, Kelantan, Malaysia

^4^Department of Family Medicine, School of Medical Sciences, Universiti Sains Malaysia, 16150 Kubang Kerian, Kelantan, Malaysia

^5^King Fahd Medical Research Center, King Abdulaziz University, Jeddah, Saudi Arabia

^6^Enzymoics, 7 Peterlee Place, Hebersham, NSW 2770, Australia

^7^Novel Global Community Educational Foundation, Australia

^8^Division of Medical Sciences & Graduate Entry Medicine, School of Medicine, University of Nottingham, Royal Derby Hospital Centre, Derby, UK

^9^School of Pharmacy, Monash University Malaysia, Jalan Lagoon Selatan, 47500 Bandar Sunway, Selangor, Malaysia

*Joint corresponding authors: Fahmida Alam ([alam.fahmida@yahoo.com](mailto:alam.fahmida@yahoo.com)) and Siew Hua Gan ([gan.siewhua@monash.edu](mailto:gan.siewhua@monash.edu))

| **Supplementary Table S1.** PRISMA check list | | | |
| --- | --- | --- | --- |
| **Section/topic** | **#** | **Checklist item** | **Reported on page #** |
| **TITLE** | | |  |
| Title | 1 | Identify the report as a systematic review, meta-analysis, or both. | 1 |
| **ABSTRACT** | | |  |
| Structured summary | 2 | Provide a structured summary including, as applicable: background; objectives; data sources; study eligibility criteria, participants, and interventions; study appraisal and synthesis methods; results; limitations; conclusions and implications of key findings; systematic review registration number. | 2 |
| **INTRODUCTION** | | |  |
| Rationale | 3 | Describe the rationale for the review in the context of what is already known. | 3-4 |
| Objectives | 4 | Provide an explicit statement of questions being addressed with reference to participants, interventions, comparisons, outcomes, and study design (PICOS). | 4 |
| **METHODS** | | |  |
| Protocol and registration | 5 | Indicate if a review protocol exists, if and where it can be accessed (e.g., Web address), and, if available, provide registration information including registration number. | 4 |
| Eligibility criteria | 6 | Specify study characteristics (e.g., PICOS, length of follow-up) and report characteristics (e.g., years considered, language, publication status) used as criteria for eligibility, giving rationale. | 5 |
| Information sources | 7 | Describe all information sources (e.g., databases with dates of coverage, contact with study authors to identify additional studies) in the search and date last searched. | 4 |
| Search | 8 | Present full electronic search strategy for at least one database, including any limits used, such that it could be repeated. | 4 |
| Study selection | 9 | State the process for selecting studies (i.e., screening, eligibility, included in systematic review, and, if applicable, included in the meta-analysis). | 5 |
| Data collection process | 10 | Describe method of data extraction from reports (e.g., piloted forms, independently, in duplicate) and any processes for obtaining and confirming data from investigators. | 5 |
| Data items | 11 | List and define all variables for which data were sought (e.g., PICOS, funding sources) and any assumptions and simplifications made. | 5-6 |
| Risk of bias in individual studies | 12 | Describe methods used for assessing risk of bias of individual studies (including specification of whether this was done at the study or outcome level), and how this information is to be used in any data synthesis. | 6 |
| Summary measures | 13 | State the principal summary measures (e.g., risk ratio, difference in means). | 5-6 |
| Synthesis of results | 14 | Describe the methods of handling data and combining results of studies, if done, including measures of consistency (e.g., I^2^) for each meta-analysis. | 6-7 |
| Risk of bias across studies | 15 | Specify any assessment of risk of bias that may affect the cumulative evidence (e.g., publication bias, selective reporting within studies). | 6 |
| Additional analyses | 16 | Describe methods of additional analyses (e.g., sensitivity or subgroup analyses, meta-regression), if done, indicating which were pre-specified. | 6 |
| **RESULTS** | | |  |
| Study selection | 17 | Give numbers of studies screened, assessed for eligibility, and included in the review, with reasons for exclusions at each stage, ideally with a flow diagram. | 7 |
| Study characteristics | 18 | For each study, present characteristics for which data were extracted (e.g., study size, PICOS, follow-up period) and provide the citations. | 7 |
| Risk of bias within studies | 19 | Present data on risk of bias of each study and, if available, any outcome level assessment (see item 12). | 7-8 |
| Results of individual studies | 20 | For all outcomes considered (benefits or harms), present, for each study: (a) simple summary data for each intervention group (b) effect estimates and confidence intervals, ideally with a forest plot. | 8-10 |
| Synthesis of results | 21 | Present results of each meta-analysis done, including confidence intervals and measures of consistency. | 8-10 |
| Risk of bias across studies | 22 | Present results of any assessment of risk of bias across studies (see Item 15). | 9 |
| Additional analysis | 23 | Give results of additional analyses, if done (e.g., sensitivity or subgroup analyses, meta-regression [see Item 16]). | 8-9 |
| **DISCUSSION** | | |  |
| Summary of evidence | 24 | Summarize the main findings including the strength of evidence for each main outcome; consider their relevance to key groups (e.g., healthcare providers, users, and policy makers). | 10-12 |
| Limitations | 25 | Discuss limitations at study and outcome level (e.g., risk of bias), and at review-level (e.g., incomplete retrieval of identified research, reporting bias). | 13-14 |
| Conclusions | 26 | Provide a general interpretation of the results in the context of other evidence, and implications for future research. | 14-15 |
| **FUNDING** | | |  |
| Funding | 27 | Describe sources of funding for the systematic review and other support (e.g., supply of data); role of funders for the systematic review. | 17 |

**Supplementary Text S1.** Search strategies used in electronic databases

1. **Medline via Pubmed**

((((((diabetes[Title/Abstract]) OR type 2 diabetes[Title/Abstract]) OR type 2 diabetic[Title/Abstract]) OR type 2 diabetes mellitus[Title/Abstract]) OR T2DM[Title/Abstract])) AND ((Pioglitazone[Title/Abstract]) OR Thiazolidinedione[Title/Abstract])

1. **Web of Science**

(TI=diabetes OR TI=type 2 diabetes OR TI=type 2 diabetic OR TI=type 2 diabetes mellitus OR TI=T2DM OR TI=non-insulin dependent*) AND (TI=Pioglitazone OR TI=Thiazolidinedione)

1. **Embase**

((‘Pioglitazone' OR 'Thiazolidinedione') AND ('diabetes' OR 'type 2 diabetes' OR 'T2DM' OR 'type 2 diabetes mellitus' OR 'type 2 diabetic')).ti

1. **Scopus**

TITLE("diabetes" OR "type 2 diabetes" OR "type 2 diabetic" OR "type 2 diabetes mellitus" OR "T2DM" OR "non-insulin dependent") AND TITLE("Pioglitazone" OR "Thiazolidinedione")

1. **ScienceDirect**

TITLE("diabetes" OR "type 2 diabetes" OR "type 2 diabetic" OR "type 2 diabetes mellitus" OR "T2DM" OR "non-insulin dependent") AND TITLE("Pioglitazone" OR "Thiazolidinedione")

1. **ClinicalTrials.gov**

Studies With Results | Interventional Studies | Type2 Diabetes | Pioglitazone ([**https://clinicaltrials.gov/ct2/results?term=&type=Intr&rslt=With&age_v=&gndr=&cond=Type2%20Diabetes&intr=Pioglitazone&titles=&outc=&spons=&lead=&id=&cntry1=&st**](https://clinicaltrials.gov/ct2/results?term=&type=Intr&rslt=With&age_v=&gndr=&cond=Type2%20Diabetes&intr=Pioglitazone&titles=&outc=&spons=&lead=&id=&cntry1=&st))

1. **Cochrane database**

| #1 | Pioglitazone:TI,AB,KY |
| --- | --- |
| #2 | Thiazolidinedione:TI,AB,KY |
| #3 | MESH DESCRIPTOR Diabetes Mellitus, Type 2 EXPLODE ALL TREES |
| #4 | #1 OR #2 |
| #5 | #3 AND #4 |

**Supplementary Text S2.** List of eligible studies excluded due to unusable data presentation.

1. Pavo, I. *et al.* Effect of pioglitazone compared with metformin on glycemic control and indicators of insulin sensitivity in recently diagnosed patients with type 2 diabetes. *J. Clin. Endocrinol. Metab.* **88**, 1637-1645 (2003).

2. Tan, M. H. *et al.* Comparison of pioglitazone and gliclazide in sustaining glycemic control over 2 years in patients with type 2 diabetes. *Diabetes Care* **28**, 544-550 (2005).

**(a)**

**(b)**

**Supplementary Figure S1.** Forest plots showing effects of pioglitazone monotherapy versus comparator monotherapies on body weight and HOMA-IR as secondary outcomes. Weighted mean difference in change from baseline in body weight **(a)** and HOMA-IR **(b)**.

**(a)**

**(b)**

**Supplementary Figure S2.** Subgroup analysis of HbA1c **(a)** and FBS **(b)** outcomes based on specific comparator drugs.

**(a)**

**(b)**

**Supplementary Figure S3.** Subgroup analysis of HbA1c **(a)** and FBS **(b)** outcomes based on geographical location.

**(a)**

**(b)**

**Supplementary Figure S4.** Subgroup analysis of HbA1c **(a)** and FBS **(b)** outcomes based on trial duration.

**(a)**

**(b)**

**Supplementary Figure S5.** Subgroup analysis of HbA1c **(a)** and FBS **(b)** outcomes based on diabetes duration.

**(a)**

**(b)**

**Supplementary Figure S6*.*** Subgroup analysis of HbA1c **(a)** and FBS **(b)** outcomes based on pioglitazone dose.

**(a)**

**(b)**

**Supplementary Figure S7.** Sensitivity analysis of HbA1c **(a)** and FBS **(b)** outcomes excluding multicentre studies.

**(a)**

**(b)**

**Supplementary Figure S8.** Sensitivity analysis of HbA1c **(a)** and FBS **(b)** outcomes excluding open-label studies.

**(a)**

**(b)**

**Supplementary Figure S9.** Sensitivity analysis of HbA1c **(a)** and FBS **(b)** outcomes excluding studies with overall high risk of bias.

**(a)**

**(b)**

**Supplementary Figure S10.** Sensitivity analysis of HbA1c **(a)** and FBS **(b)** outcomes excluding studies presented data with adjusted mean difference.

**(a)**

**(b)**

**Supplementary Figure S11.** Forest plots showing effects of pioglitazone monotherapy versus comparator monotherapies on the safety outcomes. Weighted mean difference in change from baseline in lipid parameters **(a)** and blood pressure **(b)**.

**(a)**

**(b)**

**(c)**

**Supplementary Figure S12.** Risk of adverse events with pioglitazone monotherapy versus comparator monotherapies. Adverse events are shown in **(a)**, **(b)** and **(c)** forest plots.
